# Different In Vitro Drug Susceptibility Profile of *Plasmodium falciparum* Isolates from Two Adjacent Areas of Northeast Myanmar and Molecular Markers for Drug Resistance

**DOI:** 10.3390/tropicalmed7120442

**Published:** 2022-12-17

**Authors:** Mengxi Duan, Yao Bai, Shuang Deng, Yonghua Ruan, Weilin Zeng, Xiaosong Li, Xun Wang, Wei Zhao, Hui Zhao, Kemin Sun, Wenya Zhu, Yiman Wu, Jun Miao, Myat Phone Kyaw, Zhaoqing Yang, Liwang Cui

**Affiliations:** 1 Department of Pathogen Biology and Immunology, Kunming Medical University, Kunming 650500, China; 2 Department of Pathology, Kunming Medical University, Kunming 650500, China; 3 Department of Internal Medicine, Morsani College of Medicine, University of South Florida, 3720 Spectrum Boulevard, Suite 304, Tampa, FL 33612, USA; 4 Myanmar Health Network Organization, Yangon 11181, Myanmar

**Keywords:** *Plasmodium falciparum*, the China–Myanmar border, drug resistance, in vitro assay, genetic mutations, regional difference

## Abstract

The Greater Mekong Subregion (GMS) is the epicenter of antimalarial drug resistance. We determined in vitro susceptibilities to 11 drugs of culture-adapted *Plasmodium falciparum* isolates from adjacent areas (Laiza and Muse) along the China–Myanmar border. Parasites from this region were highly resistant to chloroquine and pyrimethamine but relatively sensitive to other antimalarial drugs. Consistently, the Dd2-like *pfcrt* mutations were fixed or almost fixed in both parasite populations, and new mutations mediating piperaquine resistance were not identified. Similarly, several mutations related to *pfdhfr* and *pfdhps* were also highly prevalent. Despite their geographical proximity, malaria parasites from Laiza showed significantly higher in vitro resistance to artemisinin derivatives, naphthoquine, pyronaridine, lumefantrine, and pyrimethamine than parasites from Muse. Likewise, the *pfdhfr* N51I, *pfdhps* A581G, *pfmrp1* H785N, and *pfk13* F446I mutations were significantly more frequent in Laiza than in Muse (*p* < 0.05). For the *pfmdr1* mutations, Y184F was found only in Laiza (70%), whereas F1226Y was identified only in Muse (31.8%). Parasite isolates from Laiza showed a median RSA value of 5.0%, significantly higher than the 2.4% in Muse. Altogether, *P. falciparum* parasite populations from neighboring regions in the GMS may diverge substantially in their resistance to several antimalarial drugs. This information about different parasite populations will guide antimalarial treatment policies to effectively manage drug resistance during malaria elimination.

## 1. Introduction

The Greater Mekong Subregion (GMS) refers to six countries and regions in the Mekong River Basin, including Cambodia, Vietnam, Laos, Myanmar, Thailand, and China’s Yunnan and Guangxi provinces. China achieved the goal of malaria elimination in 2021, while the rest of the countries are trying to eliminate malaria by 2030 [1,2,3]. Myanmar has the highest malaria burden among the GMS countries [4]. Political instability and military conflict in Myanmar have driven hundreds of thousands of citizens into resettlement camps along its border, where malaria transmission is high [5]. While the camps’ poor sanitation and makeshift structures are conducive to malaria transmission, massive population movements to these camps have also contributed to malaria introduction [6]. Furthermore, intensive malaria transmission at the borders also poses a major threat to malaria introduction to neighboring countries [7]. Therefore, malaria surveillance, a core intervention promoted by the World Health Organization’s Global Technical Strategy for Malaria 2016–2030, must be strengthened to prevent the cross-border spread of malaria.

The GMS is a breeding ground for multidrug-resistant *Plasmodium falciparum*, which has developed resistance to almost all commonly used antimalarials [8]. Due to the difference in the adoption of drug policies, the epidemiological backgrounds of *P. falciparum* and *P. vivax* malaria, the public health infrastructure, and accessibility to treatment in the GMS countries, drug resistance exhibits considerable geographical variations. Even *P. falciparum* populations from closely located transmission “pockets” separated by malaria-free zones can display distinct resistance phenotypes [9]. This geographical difference highlights the necessity of resistance surveillance and management in multiple sentinel sites of malaria endemicity.

The Laiza town of Kachin State is in northeastern Myanmar and borders China. Throughout Myanmar’s history, this region has been relatively independent and has had little communication with the central government. Therefore, it has adopted a malaria prevention and treatment policy similar to that in China. Per local government policy, dihydroartemisinin–piperaquine (DHA-PPQ) has been used as the major artemisinin-based combination therapy (ACT) to treat *falciparum* malaria [10]. In addition, other antimalarial drugs, such as naphthoquine (NQ) and pyronaridine (PND), were also available for malaria treatment in the private sector. Muse is a border city of Shan State, Myanmar, 125 km south of Laiza. In recent years, economic trade between China and Myanmar has increased population movement to this region. As a result, malaria in this area is more migration-related. Malaria treatment here follows Myanmar’s antimalarial treatment policy [11], with artemether–lumefantrine designated as the first-line therapy for uncomplicated *P. falciparum* malaria [12]. Since we have installed malaria surveillance in these border areas, we were interested in determining whether *P. falciparum* parasites collected from the two border areas represent different parasite populations. After adapting clinical parasites to long-term in vitro culture, we profiled their in vitro susceptibilities to a panel of antimalarials and genotyped their drug resistance genes. This study revealed significant differences in drug resistance between these parasite populations, even though they were collected from two adjacent regions, emphasizing the importance of monitoring the sources of parasites at the ports of introduction.

## 2. Materials and Methods

### 2.1. Parasite Sample Collection

Clinical isolates of *P. falciparum* were collected in 2013 from two border areas of eastern Myanmar, Laiza and Muse, as part of cross-border malaria surveillance, control, and resistance monitoring efforts in western Yunnan province, China (Figure S1). Malaria was diagnosed by microscopy of Giemsa-stained blood smears from patients presenting to local clinics. After obtaining written informed consent or assent for minors, 2–5 mL of venous blood were drawn from patients with uncomplicated *P. falciparum* malaria. Blood samples were cryogenically stored in liquid nitrogen and subsequently adapted to long-term in vitro culture. The study protocol was approved by the Institutional Review Boards of local health bureaus and Kunming Medical University (Protocol code: IRB #KMC2011-01).

### 2.2. Parasite Culture and Drug Assays

Monoclonal infections were identified by genotyping three polymorphic genes *pfmsp1*, *pfmsp2*, and *pfglurp*, and were culture-adapted and used for in vitro drug assay and genotyping [13]. Frozen samples were thawed in solutions of NaCl and glucose. *P. falciparum* parasite isolates were cultured in RPMI 1640 culture medium with 6% human AB serum, supplemented with 0.5% Albumax II. *Plasmodium* isolates were cultured at 37 °C with type O^+^ human red blood cells (RBCs) under gas conditions of 3% O_2_, 5% CO_2_, and 92% N_2_ [14]. When the parasitemia reached ≥1% of the ring stage, the culture was synchronized with 5% sorbitol and diluted to 2% hematocrit and 0.5% parasitemia. Susceptibilities of the isolates to 11 antimalarial drugs, chloroquine (CQ), PPQ, mefloquine (MFQ), NQ, pyrimethamine (PY), artesunate (AS), DHA, artemether (AM), quinine (QN), lumefantrine (LMF), and PND, were determined using the SYBR Green Ⅰfluorimetric assay [15]. CQ, MFQ, QN, and PY were purchased from Sigma (St. Louis, MO, USA). PPQ was from Chongqing Kangle Pharmaceutical Co. (Chongqing, China), PND was from China Institute of Pharmaceutical and Biological Products (Beijing, China), and NQ, LMF, AS, and DHA were from Kunming Pharmaceutical Co. (Kunming, Yunnan, China). Stock solutions of CQ (3.75 μM), NQ (256 nM), PND (160 nM), and PPQ (320 nM) were prepared in distilled water. MFQ (256 nM), QN (10.24 μM), LMF (800 nM), AS, DHA, and AM (1.5 μM) were formulated in ethanol and PY in 1% acetic acid. Drugs were serially diluted and assayed in 96-well plates. Each drug concentration was assayed with three technical replicates and two biological replicates, while the 3D7 strain was included in each assay as an internal control.

### 2.3. Ring Survival Assay (RSA)

The RSA was performed as previously described [16,17,18]. Briefly, highly synchronous parasite cultures at the young ring stage (0–3h) were exposed to 700 nM DHA or 0.1% dimethyl sulfoxide (DMSO) as the control for 6 h. The drug was then washed off with RPMI 1640, and parasites were cultivated further for 66 h under standard in vitro culture conditions. At 72h after the assay initiation, survival rates were calculated in Giemsa-stained thin smears by counting the viable parasites surviving in DHA-treated versus DMSO-treated cultures. Parasite isolates demonstrating >1% survival were considered to display reduced susceptibility (or partial resistance) to artemisinin (ART) [19].

### 2.4. Sequencing of Genes Associated with Drug Resistance

*Plasmodium* DNA was extracted using the Roche High Pure PCR Template Preparation Kit. Polymorphisms in drug resistance genes were determined by PCR and sequencing. Included in the analysis are two *pfcrt* fragments covering codons 72 to 76, 220 and 356 [20]; a *pfdhfr* fragment covering codons 51, 59, 108, and 164; two *pfdhps* fragments covering sites 436, 540, 581; two *pfmdr1* fragments covering codons 86, 130, 184, 1034, 1042, and 1226; and a *pfmrp1* fragment covering codons 191, 325, 437, 785, 876, 1007, and 1390; the *pfnhe1* fragment containing the ms4760 minisatellite; and the complete sequences of the *pfk13* gene as previously reported [21,22,23]. To determine the copy numbers of the *pfmdr1* and *plasmepsin*2/3 genes, real-time PCR was performed using the DBI Bestar qPCR Master Mix (SYBR Green) in Quant Studio 6 Flex (Life technologies). The *β-tubulin* gene was used as an internal reference gene for normalization. Each sample was analyzed in three replicates. The amplification efficiency of these genes was determined using 3D7 as the calibration sample, and the *pfmdr1* gene in Dd2 was used as a multicopy control.

### 2.5. Statistical Analyses

Statistical analysis was performed using Graphpad Prism 6.0 for windows. The geometric mean of the half-maximal inhibitory concentration (IC_50_s) was calculated by curve fitting. Mann–Whitney U test was used to compare between two groups. The IC_50_ values and RSA of the clinical isolates were compared with those of the 3D7 strain using the Wilcoxon matched-pairs signed-rank test. A Chi-square test was used to compare the prevalence of the genetic variants between the two regions. Correlations between the IC_50_s of drugs were determined using Spearman’s test in the R package. Correlations between the RSA and drugs were determined using Spearman’s test.

## 3. Results

### 3.1. In Vitro Drug Susceptibilities of P. falciparum from the China–Myanmar Border

**Figure 1:**
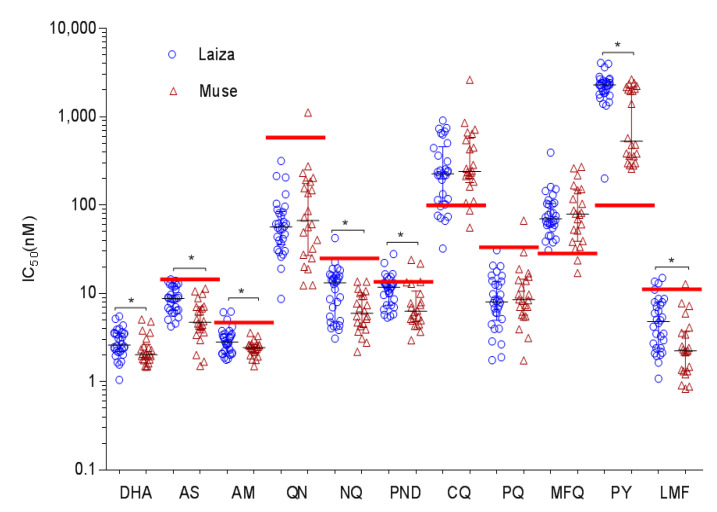
Dot plots of in vitro susceptibilities of parasite isolates from Laiza and Muse in eastern Myanmar to 11 antimalarial drugs. Asterisks indicate significant differences in drug sensitivity between the two regions (*p* < 0.05, Mann–Whitney U test). Red bars indicate the cutoff values used to define resistance: CQ, 100 nM; QN, 600 nM; PY, 100 nM; MFQ, 30 nM; PQ, 31.2 nM; LMF, 11.8 nM; AS, 14.1 nM; AM, 4.7 nM; NQ, 23.8 nM; and PND, 15 nM. DHA, dihydroartemisinin; AS, artesunate; AM, artemether; QN, quinine; NQ, naphthoquine; PND, pyronaridine; CQ, chloroquine; PPQ, piperaquine; MFQ, mefloquine; PY, pyrimethamine; LMF, lumefantrine.

We collected and cultured 52 monoclonal *P.falciparum* isolates near Laiza (30 isolates) and Muse (22 isolates), two major border checkpoints at the China–Myanmar border. In vitro susceptibilities to 11 antimalarial drugs of 52 parasite isolates and the laboratory clone 3D7 were tested using the 72 h SYBR Green I assay and RSA (Table 1, Table S1). Overall, the field parasite isolates had significantly higher IC_50_ values than 3D7 for almost all drugs (*p* < 0.05, Mann–Whitney U test) (Table 1), except for QN, LMF, and PND. For CQ, 85% of the parasites were resistant, with IC_50_ values higher than the 100 nM cutoff value (Figure 1) [24]. For MFQ, 96% of parasite isolates were considered resistant with IC_50_ above the 30 nM cutoff value proposed in an earlier study [24]. The median IC_50_ of QN was significantly lower than the cutoff value of 600 nM, as proposed earlier [24], with 2% of the isolates exceeding this value. For PND, 15% of isolates had higher IC_50_ values than the cutoff value of 15 nM, as proposed earlier [25]. Given that there were no threshold values defined for resistance to LMF, NQ, PPQ, AS, and AM, we used the mean + 2 standard deviations (SD) as arbitrary cutoff values for potential resistance [26]. For LMF, 8% isolates had higher IC_50_ values above 11.8 nM. For AM, 6%of isolates had higher IC_50_ values above 4.7nM. For NQ, PPQ, and AS, 2% of isolates had higher IC_50_ values than the cutoff values (Figure 1). For the antifolate drug PY, IC_50_ ≤ 100 nM, 100 < IC_50_ ≤ 2000 nM, and IC_50_ ≥ 2000 nM were classified as sensitive, moderately resistant, and highly resistant, respectively [24,27]. According to this classification scheme, 52% of isolates were considered highly resistant to PY (IC_50_s ≥ 2000 nM), and 48% of isolates tested were considered moderately resistant to PY (100 < IC_50_ ≤ 2000 nM) (Figure 1). Although the parasites were relatively susceptible to most of the drugs tested, the IC_50_ values of individual parasite isolates varied widely. For example, the IC_50_ values between the least and most susceptible isolates differed by 38-fold for PPQ, ~19-fold for NQ and LMF, 23-fold for MFQ, and >127-fold for QN (Table 1). These results indicated the existence of parasite isolates with substantially reduced susceptibility to many antimalarial drugs, although clinical resistance was not clear. RSA values of the 52 clinical isolates were significantly higher than that for 3D7 (Table 1). The range of the RSA values for the field isolates was relatively wide (0–14.7%). Using 1% RSA value as the cutoff value for partial ART resistance, 49 (94%) parasite isolates had RSA values greater than 1%.

### 3.2. Correlations between Susceptibilities to Different Drugs

**Figure 2:**
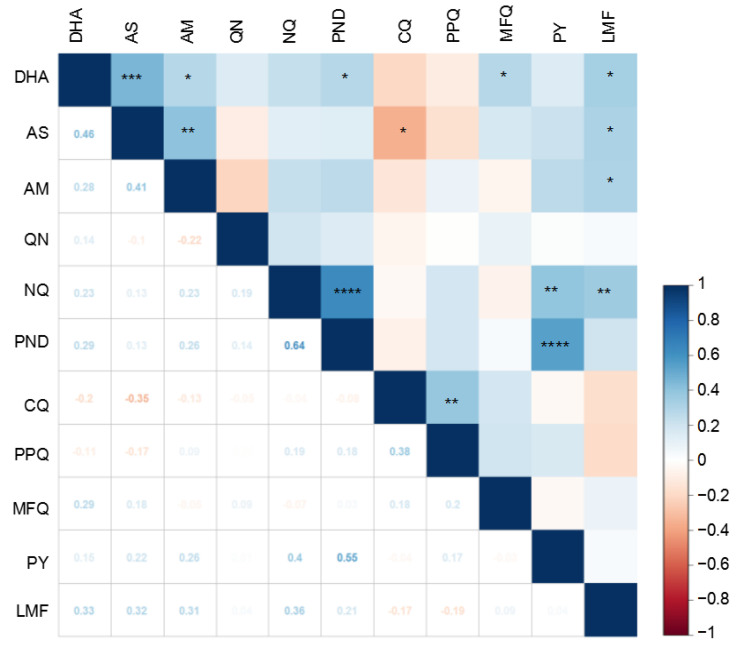
Correlation between the IC_50_ values of cultured parasite isolates to 11 antimalarial drugs by Spearman’s test. The degree of correlation is color-coded, and coefficients are shown below the diagonal. *, **, ***, and **** indicate significance at 0.01 < *p* < 0.05, 0.001 < *p* < 0.01, 0.0001 < *p* < 0.001, and *p* < 0.0001, respectively. Abbreviations are the same as in Figure 1.

Consistent with a similar mode of action for the ART derivatives, the susceptibilities of the parasites to DHA, AS, and AM were positively correlated (*p* < 0.05) (Figure 2). Interestingly, the pairwise comparison revealed a significant positive correlation between PND and NQ or PY (*p* < 0.0001, Spearman’s test). As we found in an earlier study [28], parasites from eastern Myanmar showed a significant positive correlation between the two 4-aminoquinoline drugs, CQ and PPQ (*p* < 0.01, Spearman’s test). In addition, there was a weak, positive correlation between DHA and PND or MFQ (*p* < 0.05). Susceptibility to LMF was also weakly correlated with those of the three ART derivatives (*p* < 0.05). Furthermore, there was a moderate, positive correlation between NQ and PY or LMF (*p* < 0.01). Finally, the only significant negative correlation was identified between CQ and AS (*p* < 0.05). Pairwise comparison between the RSA and IC_50_ values for both AS and DHA showed no significant correlation (*p* > 0.05, Spearman’s correlation test) (Figure S2), except for AM (*p* < 0.05, Spearman’s correlation test), further indicating that the two assays measured different resistance phenotypes (Figure S2).

### 3.3. Polymorphisms in Genes Associated with Drug Resistance

We sequenced seven genes related to drug resistance (*pfdhfr*, *pfdhps*, *pfcrt*, *pfmdr1*, *pfmrp1*, *pfnhe1*, and *pfk13*) in all isolates and identified mutations by sequence alignment with 3D7. We performed statistical analysis to determine whether certain *pfk13* mutations were associated with the RSA phenotypes.

***Pfdhfr* and *pfdhps***. The three mutations C59R, S108N, and I164L in *pfdhfr* mediating resistance to PY were fixed or approached fixation in the parasite population (Table 2). The N51**I**mutation also reached a high prevalence of 73.1%. Parasites carrying quadruple mutations (**IRNL**) reached 73.1%, suggesting high resistance to PY (Table 3). Consistent with the fact that the *pfdhfr* mutations mediate resistance to PY, both the N51**I** and I164**L** mutations were associated with significantly elevated in vitro IC_50_s to PY (Figure S3). Similarly, parasites carrying the quadruple mutations **IRNL** were also significantly more resistant to PY than those with triple (N**RNL**) or double mutations (N**RN**I) (Figure S3). In *pfdhps*, K540E/N was nearly fixed, while two other mutations, S436A and A581G, reached relatively high levels (>48%). Parasites carrying double mutations for these three positions accounted for 90.4% (Table 3).

***Pfcrt***. Consistent with widespread resistance to CQ in the GMS, the M74I, N75E, K76T, A220S, and I356T mutations were all fixed or approached fixation in the parasite populations. However, while parasites collected before 2013 were all wild-type at C72 [29], ~20% of parasites collected in 2013 carried the C72S mutation. As a result, the *pfcrt* CVIET haplotype (positions 72 to 76) decreased to 79% in 2013. Interestingly, although the C72S mutation did not change the parasites’ susceptibility to CQ, the **SIETS** haplotype showed significantly increased susceptibility to LMF and NQ but significantly decreased susceptibility to QN (*p* < 0.05, Mann–Whitney U test) (Figure S4).

***Pfmdr1***. We detected six mutations in the *pfmdr1* gene (occurring at 2–40%). Y184F was the most frequent (40%), followed by F1226Y (14%) (Table 2). The N86Y mutation was relatively rare (1.9%). For the two most frequent mutations, Y184F was associated with increased in vitro resistance to LMF, AS, and MFQ, while F1226Y was related to increased susceptibility to PND and NQ (*p* < 0.05, Mann–Whitney U test) (Figure S5). The two most abundant *pfmdr1* haplotypes were the wild-type (36.5%) and NE**F**SNF with a single Y184F mutation (40.4%). Using real-time PCR and Dd2 parasite as a multicopy *pfmdr1* reference, we found that all *Plasmodium* isolates had single-copy *pfmdr1* (data not shown).

***Pfmrp1***. Seven mutations were detected in *pfmrp1* with frequencies ranging from 5.8% to 90.4%. Five mutations, H191Y, S437A, H785N, I876V, and T1007M, had >60% prevalence in the parasite population (Table 2). The correlation between *pfmrp1* mutations and in vitro drug susceptibilities is complex. Of the eight mutations associated with altered drug responses, three correlated with reduced susceptibility. The H191Y and S437A mutations were associated with increased in vitro resistance to MFQ, while N325S was correlated with increased resistance to PPQ. While the I876V mutation was associated with increased susceptibility to PND, NQ, and PPQ, F1390I was correlated with increased sensitivity to the ART drugs DHA and AS. In addition, H785N and T1007M were associated with increased susceptibility to PPQ (Figure S6). Parasites carrying quintuple mutations **Y**N**ANVM**F reached almost 70% (Table 3), and this haplotype showed a much higher IC_50_ value for MFQ than the wild-type HNSHITF (*p* < 0.05, Mann–Whitney U test, not shown).

***Pfnhe1***. The minisatellite ms4760 allelic variants in the *pfnhe1* gene were associated with QN resistance in some *Plasmodium* populations [9,30]. Five variants were identified in the parasites (Table 3), three of which, MS-5 (40.4%), MS-7 (34.6%), and MS-6 (17.3%), accounted for more than 90% of the parasite population (Table 3). Whereas only the rare haplotype MS-3 of *pfnhe1* was associated with increased resistance to QN, both MS-5 and MS-6 showed reduced susceptibility to AS and AM. MS-6 was also correlated with decreased susceptibility to PND (*p* < 0.05, Mann–Whitney U test, Figure S7).

***Pfk13***. Three *pfk13* point mutations (K189T, F446I, and N458Y) were detected in the parasite samples, and two were located in the propeller domain (>440 amino acids). F446I remained the predominant mutation (63.5%), which was associated with reduced susceptibility to both DHA and AS (Figure S8). In addition, 88.5% of *Plasmodium* parasites contained the NN insertion in the N-terminal region between positions 137 and 142 (Table 2). Since mutations in the propeller domain of the K13 protein (>440 amino acids) were associated with clinical ART resistance [31], we classified 52 parasites into two groups based on the presence or absence of mutations in the propeller domain. Parasites with mutations in the propeller domain showed a median RSA value of 4.9%, significantly higher than the 2.1% in the K13 WT group (*p* < 0.05, Mann–Whitney U test, Figure S9a). The most common mutation, F446I (33 isolates), also showed significantly higher median RSA values of 4.9% (*p* < 0.05, Mann–Whitney U test, Figure S9b, Table S2) than the WT. We also found that parasites from Muse with the K189T mutation located outside the Kelch domain showed relatively high RSA values, with the median value being significantly higher than that of the WT isolates (*p* < 0.05, Mann–Whitney U test, Figure S9b, Table S2). Moreover, parasites from Muse with the N458Y mutation had a higher median RSA (5.0%) than parasites from the K13 WT group (2.1%), although the difference was not significant (*p* > 0.05, Mann–Whitney U test, Figure S9b, Table S2).

### 3.4. Multidrug Resistance Haplotypes

The clonality of the parasite isolates allows us to assess the multidrug resistance (MDR) haplotypes. We focused our attention on haplotypes of the following five genes with well-defined resistance to 4-aminoquinolines (*pfcrt* and *pfmdr1*), antifolate drugs (*dhfr*/*dhps*), and artemisinins (K13). This analysis revealed that >65.4% (34/52) of parasite isolates carried MDR haplotypes (*pfcrt* 76**T**, quintuple–sextuple mutations in *dhfr*/*dhps*, and K13 446**I** or 458**Y**) that would confer resistance to 4-aminoquinoline, antifolate, and artemisinin drugs (Figure S10). Fifteen (28.9%) isolates carried the *pfcrt* 76**T** and quintuple–sextuple mutations in *dhfr*/*dhps* but lacked K13 mutations, suggesting resistance to 4-aminoquinolines and antifolates. Only two parasites had *pfcrt* 76**T** and quadruple mutations in *dhfr*/*dhps*, suggesting lower-level resistance to antifolate drugs.

### 3.5. Regional Differences in Drug Susceptibility and Genetic Polymorphisms

*Plasmodium* isolates from these two regions had different susceptibilities to seven of the 11 antimalarial drugs tested. Parasite isolates from Laiza (the northern site) had significantly higher IC_50_s for AS, DHA, AM, NQ, PND, PY, and LMF than parasites from Muse (the southern site) (*p* < 0.05, Mann–Whitney U test, Figure 1, Table 1). Notably, the median IC_50_ of parasites to PY from Laiza was more than 4-fold higher than that from Muse (Table 1). Meanwhile, the RSA values varied by region. Parasite isolates from Laiza (the northern site) showed a median RSA value of 5.0%, significantly higher than the 2.4% in Muse (the southern site) (*p* = 0.0495, Mann–Whitney U test, Figure S11).

The two parasite populations also showed different prevalences in mutations of resistance-related genes (Table 2). For genes involved in antifolate resistance, the N51I mutation was significantly more prevalent in Laiza (96.7%) than Muse (40.9%) (*p* < 0.05). As a result, the *pfdhfr* haplotype with quadruple mutations (**IRNL** at positions 51/59/108/164) was significantly more abundant in Laiza. The S436A mutation in *pfdhps* was significantly more prevalent in Muse (68.2%) than Laiza (33.3%), whereas A581G showed the opposite trend (*p* < 0.01, χ^2^ test, Table 2). Likewise, the most abundant haplotypes S**NG** and **AE**A (at positions 436/540/581) also showed contrasting prevalence at the two sites (Table 3).

For *pfcrt*, the C72S mutation was more common in the Muse samples (36.4%) (Table 2), which resulted in the more prevalent **SIEVTS** haplotype (at positions 72/74/75/76/220) in Muse (Table 3). For *pfmdr1*, two mutations showed different frequencies between the two sites. The Y184F mutations were only found in the Laiza parasites (70%), whereas the F1226Y mutation (31.8%) was only present in the Muse population (Table 2). For the *pfmrp1* gene, only the H785N mutation was more prevalent in Laiza than Muse (*p* < 0.01, χ^2^ test).

For the *pfnhe1* gene, the two most abundant haplotypes (MS-5 and MS-7) showed contrasting prevalence in the two regional populations (Table 3). For the *pfk13* gene, although the F446I mutation was predominant in both sites, it was at a much higher frequency in Laiza (86.7%) than Muse (31.8%). Interestingly, two additional mutations, K189T and N458Y, were only present in the Muse population (Table 2).

For the MDR haplotypes, Laiza and Muse also differed drastically (Figure S10). Laiza had a significantly higher proportion of MDR parasites than Muse (86.7% vs.36.4%; *p* < 0.05, χ^2^ test). Conversely, Muse had a much higher percentage of parasites resistant to 4-aminoquinoline and antifolates than Laiza (50% vs.13.3%). Only Muse had parasites resistant to 4-aminoquinoline drugs but less resistant to antifolates (with quadruple mutations in *dhfr*/*dhps*). These results collectively indicated that parasites from Laiza had a relatively higher level of MDR parasites.

## 4. Discussion

Multidrug-resistant *P. falciparum* is a significant challenge to the global efforts of malaria eradication. Although *P. falciparum* incidence in the GMS has continually declined, monitoring drug resistance has remained critical for updating regional antimalarial drug policies. This study represents our efforts to monitor drug resistance in sentinel sites along the China–Myanmar border using in vitro drug assays and molecular surveillance. By assessing the in vitro susceptibilities of parasites from two adjacent but separated areas to 11 common antimalarial drugs, we identified their significantly divergent drug susceptibility profiles, with parasites from Laiza having significantly higher IC_50_s to NQ, PY, AS, DHA, AM, PND, and LMF than those from Muse. Consistent with the in vitro phenotypic difference, parasites from the two regions were also distinctive in resistance-conferring mutations, possibly reflecting different origins of parasite populations and different drug histories.

CQ and antifolate drugs have long been withdrawn from treating *falciparum* malaria, but parasites from the two regions remained highly resistant to these drugs. Consistent with this, resistance-conferring mutations in *pfcrt*, *pfdhfr*, and *pfdhps* were highly prevalent, with many remaining fixed in the parasite populations. Persistent CQ resistance has been speculated to be due to continued CQ selection pressure from the widespread use of CQ to treat sympatric *P. vivax* infections [29] and fixation of mutations in *pfcrt* that mediate CQ resistance in the parasite populations [32]. Since parasites from the hypoendemic regions are predominantly monoclonal, and intrahost competition is low, these mutations are likely preserved even though they inflict high fitness costs. In comparison, the *pfmdr1* N86Y mutation, which was also associated with CQ resistance [33], was infrequent in the GMS parasite populations. On the other hand, the persistent resistance to antifolates may be related to their use to treat bacterial infections [34,35,36]. For example, trimethoprim–sulfamethoxazole, used to treat acute respiratory infections, presents cross-resistance with pyrimethamine and sulfadoxine [34,35].Differential use of antifolate drugs in Laiza and Muse may account for the >4-fold differences in PY susceptibility and different frequencies of key mutations in *pfdhfr* (N51I) and *pfdhps* (S436A and A581G) between the two populations.

Partial artemisinin resistance, displayed as delayed parasite clearance following treatment with an ACT, has been detected in all the GMS regions, and the K13-propeller mutations have been used widely to track the emergence and spread of ART-resistant *P. falciparum* [31,37]. Within the GMS, *pfk13* mutations are diverse and region-specific [38,39]. F446I was most prevalent in northern Myanmar and the China–Myanmar border [38,40], and this study further confirmed this. Although the N-terminal NN insertion was associated with altered susceptibility to ART drugs [41] and its prevalence has increased dramatically over the years along the China–Myanmar border [42], we did not identify the association of this insertion with changes in in vitro IC_50_ values to DHA and AS. Siddiqui et al. showed that the N458Y mutation, which occurs at the China–Myanmar border, confers ART resistance with a significant increase in RSA values in vitro [43]. In our study, the median RSA was higher in parasites with the N458Y mutation (5.0%) than in the K13 WT group (2.1%), but the difference was not significant, probably because the number of samples with the N458Y mutation was limited and therefore it was difficult to draw firm conclusions about the association of certain K13 genotypes with reduced ART susceptibility. Consistent with previous reports [43], F446I is associated with significantly higher RSA values than the WT parasites. Globally, K189T was identified at a relatively high proportion in the Amazon basin [44]. This mutation showed a similar prevalence in Africa, but was rarely described in Southeast Asia [45,46,47,48]. The study by Wu et al. in 2020 showed that K189T was first discovered in Myanmar [49]. Reports showed that K189T mutation is associated with delayed parasite clearance, but there is no clear correlation with ART resistance [48,50,51]. However, in our study, the RSA values of the samples with K189T mutation were significantly higher than those of WT type, which provides a direction for future studies.

In Cambodia, AS-MFQ was the first ACT introduced, but its efficacy steadily declined in the early 2000s [52], resulting in the switch to DHA-PPQ in 2008. However, with partial ART resistance emerging in the region, this ACT was soon found to be ineffective [53,54]. The concurrent recovery of the sensitivity of the parasites to AS-MFQ led to the consideration of recycling this ACT. ACT failures in Cambodia appear largely due to resistance to partner drugs. MFQ and PPQ seem to impose opposing selection on drug targets, especially *pfmdr1* copy number: MFQ is associated with *pfmdr1* amplification, whereas PPQ selects single-copy *pfmdr1*. Such opposite selection on the same target has been the basis for including these drugs in a triple ACT design [55]. The main ACT used in the China–Myanmar border area was DHA-PPQ, which remained highly efficacious [10,56]. Our recent study using an in vitro drug assay also showed that parasites from the China–Myanmar border area were largely susceptible to PPQ [57]. Consistently, parasites from this region did not contain *plasmepsin*2/3 amplification [58,59] or the new *pfcrt* mutations (H97Y, F145I, M343L, and G353V) conferring PPQ resistance [60,61,62], while the parasites also contained predominantly single-copy *pfmdr1*. It is noteworthy that the I356T mutation fixed in the study populations was significantly associated with decreased QN sensitivity and increased MFQ sensitivity in *P. falciparum* parasites from Africa [20]. In addition, I356T and N326S might be the background mutations on which *pfk13* mutations emerged [63].

The ATP-binding cassette (ABC) transporters, including *pfmdr1* and *pfmrp1*, are involved in parasite resistance to multiple antimalarial drugs [64]. The Y184F mutation was only detected in Laiza, and the F1226Y mutation in Muse. The Y184F mutation was associated with increased resistance to AS, LMF, and MFQ, while the F1226Y mutation was correlated with increased susceptibility to PND and NQ, suggesting that the divergent *pfmdr1* mutations may be partially responsible for the differences in drug susceptibility between the two neighboring sites. MFQ has not been deployed in the China–Myanmar border area. Consistently, parasites from the two areas showed similar sensitivity to MFQ and *pfmdr1* amplification associated with MFQ resistance was not detected [65]. While this and an earlier study identified that several mutations in *pfmrp1* were associated with altered sensitivities to a number of drugs [23], they were similarly prevalent in the two populations. Thus, the significance of the *pfmrp1* mutations in the context of *pfcrt* and *pfmdr1* haplotypes warrants further investigation.

Antimalarial therapy is one of the pillars of malaria control and elimination. Updated knowledge about antimalarial resistance in malaria parasites is critical for delivering effective frontline treatment. Myanmar has the heaviest malaria burden in the GMS and is also a gateway to the Indian subcontinent. Thus, effective management of border malaria is essential for preventing cross-border spillover of resistant parasites, especially to neighboring countries that have just become malaria-free. This and an earlier study have demonstrated drastic differences in drug resistance between neighboring parasite populations during the elimination phase in the GMS [9]. A limitation of this study is the relatively small sample size. As malaria incidence has plummeted in recent years, collecting laboratory culture samples has become difficult. Differences in anthropology, administration, public health infrastructure, access to treatment, and intensity of malaria transmission across borders may contribute to the observed differences in susceptibility to antimalarial drugs. This may require timely adjustment of the antimalarial drug policy between different strata of malaria endemicity.

## Figures and Tables

**Table 1:** In vitro susceptibilities (IC_50_ in nM) of culture-adapted field isolates from the China–Myanmar border sites to 11 antimalarial drugs.

Drug	Total (N = 52) (Mean ± SD)	3D7 Control (Mean ± SD)	*p*-Value * Field Sites vs. Control	Laiza (N = 30)		Muse (N = 22)		*p*-Value ^#^ Laiza vs. Muse
				(Mean ± SD)	Range	(Mean ± SD)	Range	Muse
CQ	358 ±394.9	17.98 ± 0.53	<0.0001	303.3 ±241.6	32.14–903.23	432.6 ±536.7	55.41–2616	0.4010
PPQ	10.98 ±10.11	5.20 ± 0.26	<0.0001	9.76 ±6.58	1.75–30.71	12.64 ±13.55	1.74–66.65	0.5847
MFQ	93.49 ±69.44	15.40 ± 0.37	<0.0001	89.51 ±66.73	30.56–391.87	98.92 ±74.22	16.99–269.53	0.8869
NQ	9.93 ±6.94	5.36 ±2.39	<0.0001	12.17 ±8.01	3.08–42.31	6.88 ±3.41	2.18–13.59	0.0053
PY	1796 ±989.4	66.73 ± 1.28	<0.0001	2259 ±763.8	199.97–4058.0	1166 ±922	258.3–2640.0	0.0003
AS	7.46 ±3.34	5.43 ± 2.01	0.0002	8.92 ±2.98	4.22–14.37	5.48 ±2.78	1.51–11.32	0.0001
DHA	2.70 ±1.05	0.73 ± 0.13	<0.0001	2.90 ±1.04	1.05–5.49	2.42 ±1.02	1.48–5.05	0.0319
AM	2.79 ±0.96	1.36 ± 1.59	<0.0001	3.06 ±1.13	1.77–6.19	2.43 ±0.48	1.5–3.58	0.0244
QN	103.6 ±159.9	86.75 ± 1.91	0.2790	74.59 ±65.5	8.65–314.7	143.1 ±230.9	12.33–1113	0.5481
LMF	4.58 ±3.60	4.60 ± 2.90	0.4193	5.60 ±3.77	1.08–14.93	3.18 ±2.89	0.83–12.71	0.0038
PND	10.27 ±5.445	8.51 ± 1.38	0.0533	11.69 ±4.96	5.31–27.87	8.34 ±5.59	2.94–23.88	0.0031
RSA ^a^	3.83% (1.83–7.26%)	0.3%	<0.0001	4.99% (1.87–8.4)	2.4% (1.72–3.79)	0.0495

* Comparison between total field isolates and 3D7 by the Wilcoxon matched-pairs signed-rank test. ^#^ Comparison between the two sites by the Mann–Whitney U test. ^a^ The ring survival assay (RSA) values are shown as median (interquartile range). Abbreviations: SD, standard deviation; CQ, chloroquine; PPQ, piperaquine; MFQ, mefloquine; NQ, naphthoquine; PY, pyrimethamine; AS, artesunate; DHA, dihydroartemisinin; AM, artemether; QN, quinine; LMF, lumefantrine; PND, pyronaridine.

**Table 2:** The prevalence of mutations (%) in genes associated with drug resistance in the two border regions.

Gene	Mutations	Total (n = 52)	Laiza (n = 30)	Muse (n = 22)	*p*-Value
*Pfdhfr*	N51**I**	73.1	96.7	40.9	<0.0001 *
	C59**R**	100	100	100	1.0000
	S108**N**	100	100	100	1.0000
	I164**L**	96.2	100	90.9	0.1742
*Pfdhps*	S436**A**	48.1	33.3	68.2	0.0238 *
	K540**E/N**	96.2	96.7	95.5	1.0000
	A581**G**	55.8	73.3	31.8	0.0046 *
*Pfcrt*	C72**S**	19.2	6.7	36.4	0.0117 *
	M74**I**	100	100	100	1.0000
	N75**E**	100	100	100	1.0000
	K76**T**	100	100	100	1.0000
	A220**S**	98.1	100	95.5	0.4231
	I356**T**	100	100	100	1.0000
*Pfmdr1*	N86**Y**	1.9	3.3	0	1.0000
	G130**K**	5.8	0	13.6	0.0697
	Y184**F**	40.4	70	0	<0.0001 *
	S1034**I**	1.9	0	4.5	0.4231
	N1042**D**	1.9	0	4.5	0.4231
	F1226**Y**	13.5	0	31.8	0.0013 *
*Pfmrp1*	H191**Y**	90.4	86.7	95.5	0.3814
	N325**S**	5.8	3.3	9.1	0.5670
	S437**A**	90.4	86.7	95.5	0.3814
	H785**N**	71.2	83.3	54.5	0.0324 *
	I876**V**	76.9	80	72.7	0.7402
	T1007**M**	73.1	83.3	59.1	0.0647
	F1390**I**	5.8	0	13.6	1.0000
*PfK13*	NN insert	88.5	96.7	77.3	0.0716
	K189**T**	3.8	0	9.1	0.1742
	F446**I**	63.5	86.7	31.8	<0.0001 *
	N458**Y**	3.8	0	9.1	0.1742

* *p* < 0.05 (χ^2^ test).

**Table 3:** The prevalence of haplotypes of resistance genes.

Gene (Codon Positions)	Haplotypes	Total (n = 52)	Laiza (n = 30)	Muse (n = 22)	*p*-Value
*Pfdhfr* (51/59/108/164)	N**RN**I	3.8	0	9.1	0.1742
	N**RNL**	23.1	3.3	50.0	0.0001 *
	**IRNL**	73.1	96.7	40.9	<0.0001 *
*Pfdhps* (436/540/581)	SKA	1.9	0	4.5	0.4231
	SK**G**	1.9	3.3	0	1.0000
	S**NG**	40.4	56.7	18.2	0.0093 *
	S**EG**	7.7	6.7	9.1	1.0000
	**AN**A	3.8	0	9.1	0.1742
	**AE**A	38.5	26.7	54.5	0.0499 *
	**AEG**	3.8	3.3	4.5	1.0000
	**ANG**	1.9	3.3	0	1.0000
*Pfcrt* (72/74/75/76/220)	C**IET**A	1.9	0	4.5	0.4231
	C**IETS**	78.8	93.3	59.1	0.0048 *
	**SIETS**	19.2	6.7	36.4	0.0117 *
*Pfmdr1* (86/130/184/1042)	NEYSNF	36.5	26.7	50.0	0.1442
	NEYSN**Y**	11.5	0	27.3	0.0037 *
	NEY**I**N**F**	1.9	0	4.5	0.4231
	NE**F**SNF	40.4	70.0	0	<0.0001 *
	N**K**YSNF	5.8	0	13.6	0.0697
	**Y**EYSNF	1.9	3.3	0	1.0000
	NEYS**DY**	1.9	0	4.5	0.4231
*Pfmrp1* (191/325/437/785/ 876/1007/1390)	HNSHITF	9.6	13.3	4.5	0.3814
	**Y**N**A**HITF	5.8	0	13.6	0.0697
	**YSA**HITF	5.8	3.3	9.1	0.5670
	**Y**N**A**H**V**T**I**	5.8	0	13.6	0.0697
	**Y**N**A**H**VM**F	1.9	0	4.5	0.4231
	**Y**N**AN**I**M**F	1.9	3.3	0	1.0000
	**Y**N**ANVM**F	69.2	80.0	54.5	0.0701
*PfK13* (189/446/458)	KFN	28.8	13.3	50.0	0.0057 *
	K**I**N	63.5	86.7	31.8	<0.0001 *
	KF**Y**	3.8	0	9.1	0.1742
	**T**FN	3.8	0	9.1	0.1742
*Pfnhe1*	MS-3	3.8	0	9.1	0.1742
	MS-5	40.4	66.7	4.6	<0.0001 *
	MS-6	17.3	26.7	4.6	0.0618
	MS-7	34.6	6.7	72.7	<0.0001 *
	MS-21	3.8	0	9.1	0.1742

* Significant difference (χ^2^ test). Letters in bold indicate mutant residues.

## Data Availability

The data for this study are presented within this article and any further information regarding this study can be reasonably requested from the corresponding author.

## Supplementary Materials

The following supporting information can be downloaded at: https://www.mdpi.com/article/10.3390/tropicalmed7120442/s1, Figure S1: Map of the China–Myanmar border sites, Laiza and Muse, where parasites were collected; Figure S2: Spearman’s correlation between RSA (%) and IC_50_ values for dihydroartemisinin, artesunate, andartemether; Figure S3: Association of SNPs and major haplotypes in *pfdhfr* with responses to different antimalarials (shown in the y-axis or below the x-axis). *Indicates significant differences (*p* < 0.05, Mann–Whitney U test) in sensitivity between parasites carrying the two alleles or haplotypes; Figure S4: Association of SNPs and major haplotypes in *pfcrt* with responses to different antimalarials (shown in the y-axis or below the x-axis). *Indicates significant differences (*p* < 0.05, Mann–Whitney U test) in sensitivity between parasites carrying the two alleles or haplotypes; Figure S5: Association of SNPs and major haplotypes in *pfmdr1* with responses to different antimalarials (shown in the y-axis or below the x-axis). *Indicates significant differences (*p* < 0.05, Mann–Whitney U test) in sensitivity between parasites carrying the two alleles or haplotypes; Figure S6: Association of SNPs and major haplotypes in *pfmrp1* with responses to different antimalarials (shown in the y-axis or below the x-axis). *Indicates significant differences (*p* < 0.05, Mann–Whitney U test) in sensitivity between parasites carrying the two alleles or haplotypes; Figure S7: Association of SNPs and major haplotypes in minisatellites in *pfnhe1* with responses to different antimalarials (shown in they-axis or below the x-axis). *Indicates significant differences (*p* < 0.05, Mann–Whitney U test) in sensitivity between parasites carrying the two alleles or haplotypes; Figure S8: Association of SNPs and major haplotypes in *PfK13* with responses to different antimalarials (shown in they-axis or below the x-axis). *Indicates significant differences (*p* < 0.05, Mann–Whitney U test) in sensitivity between parasites carrying the two alleles or haplotypes; Figure S9: Correlation of parasite survival rates (RSA_0-3h_) and K13 polymorphisms. (A) RSA values between parasites with mutations in the Kelch propeller domain (>440 mutations) and parasites without K13 mutations. Different K13 mutations were color-coded (*p* < 0.05, Mann–Whitney U test). (B) Comparison of RSA values between parasites with different K13 mutations and the wild-type (WT) (Mann–Whitney U test). ^*^, *p* < 0.05; Figure S10: Multidrug resistance haplotypes of parasites from Laiza and Muse. Mutant amino acids are in bold. The haplotypes include *Pfcrt*(76**T)**, *pfmdr1* (Y184**F**/F1226**Y**), *pfdhfr* (N51**I**/59**R**/108**N**/I164**L**), *pfdhps* (S436**A**/K540**N/E**/A581**G**), and *pfk13* (446**I** or 458**Y**;—indicates K13 WT). The four parasites with quadruple or triple *dhfr*/*dhps* mutations are highlighted in cyan and yellow color, respectively. Group 1—76T, quintuple–sextuple *dhfr*/*dhps*, and K13 mutations (MDR); Group 2—76T and quintuple–sextuple *dhfr*/*dhps*; Figure S11: Ring survival rates of parasite isolates in the two border regions. ^*^, *p* < 0.05; Table S1: In vitro sensitivities (IC_50_, nM) to 11 antimalarial drugs and mutations in genes (*pfdhfr, pfdhps, pfcrt, pfmdr1, pfmrp1, pfhne1*,and *pfk13*) associated with altered drug sensitivities of parasite isolates from Laiza and Muse. Abbreviation: dihydroartemisinin (DHA), artesunate (AS), artesunate (AM), quinine (QN), naphthoquine (NQ), pyronaridine (PND), chloroquine (CQ), piperaquine (PQ), mefloquine (MFQ), pyrimethamine (PY), lumefantrine (LMF), and ring survival assay (RSA). Table S2: Ring survival rates of parasite isolates stratified by the K13 alleles.
