# Supplementary material for: Different In Vitro Drug Susceptibility Profile of Plasmodium falciparum Isolates from Two Adjacent Areas of Northeast Myanmar and Molecular Markers for Drug Resistance
Source: Trop Med Infect Dis. 2022 Dec 17;7(12):442. doi: 10.3390/tropicalmed7120442 (PMC9782301; doi:10.3390/tropicalmed7120442)
Supplement: Supplementary file 1 [file tropicalmed-07-00442-s001.zip › Suppl. Figures.ppt]

## Slide 1
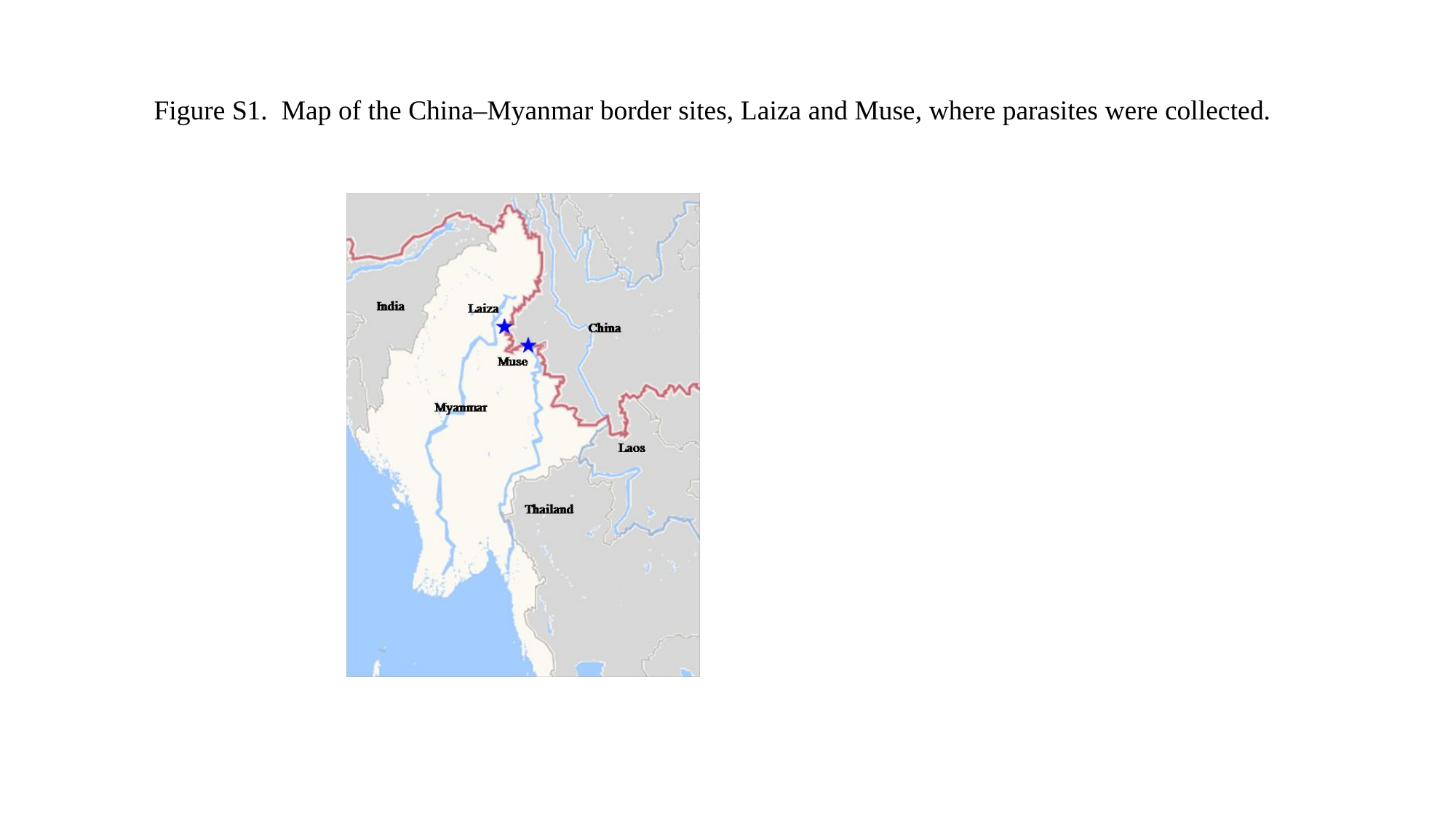

Figure S1. Map of the China–Myanmar border sites, Laiza and Muse, where parasites were collected.

## Slide 2
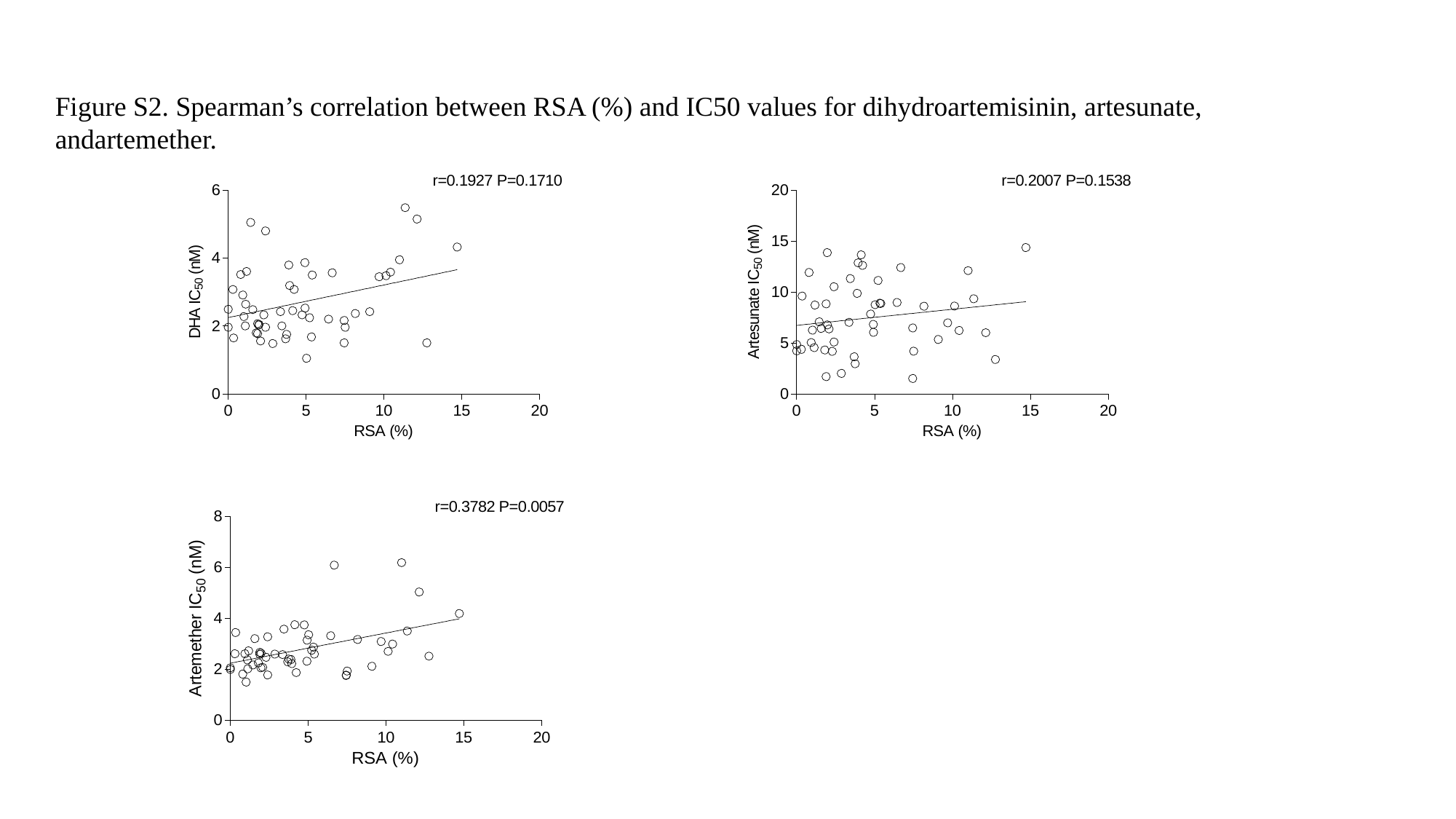

Figure S2. Spearman’s correlation between RSA (%) and IC50 values for dihydroartemisinin, artesunate, andartemether.

## Slide 3
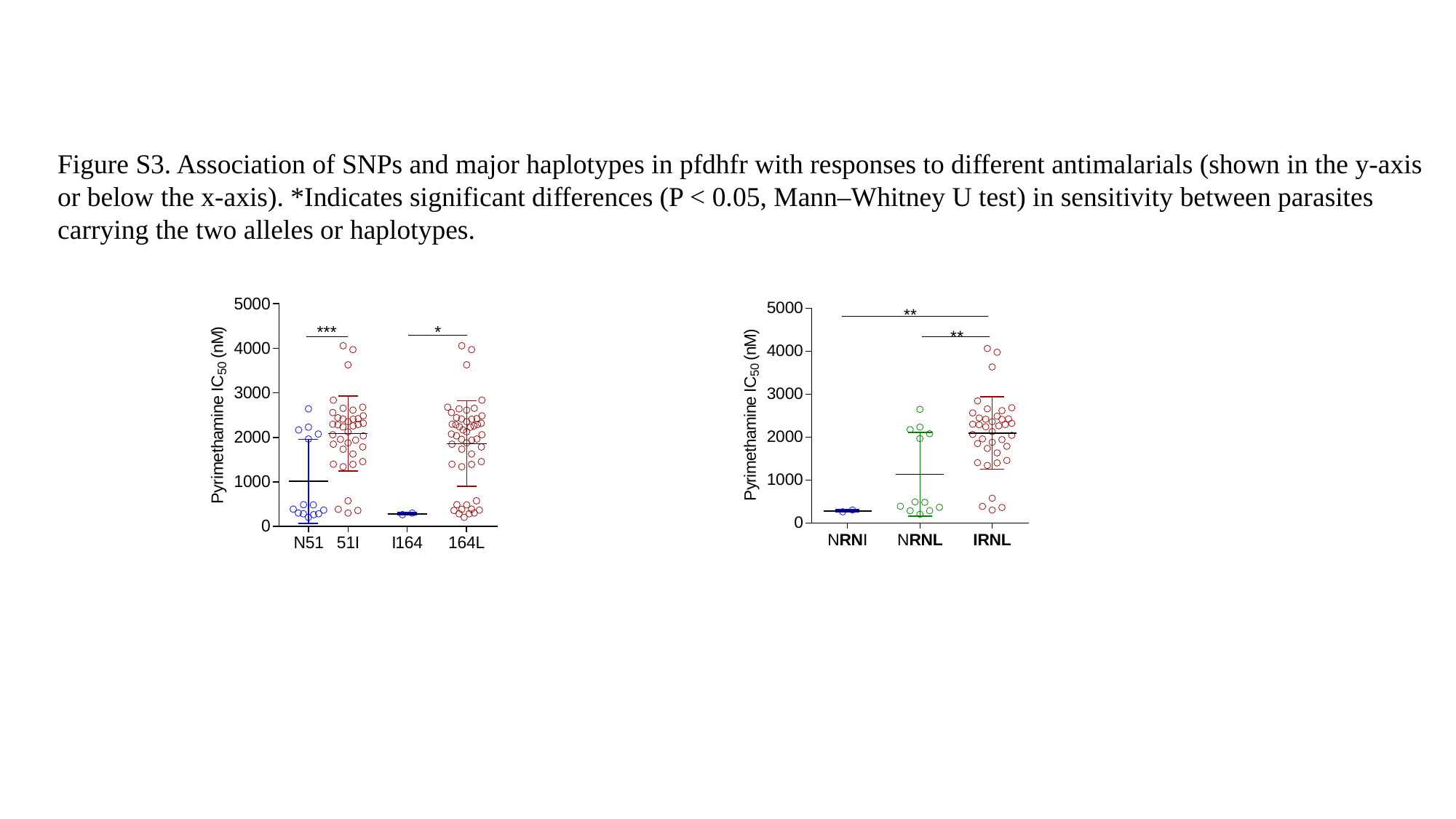

Figure S3. Association of SNPs and major haplotypes in pfdhfr with responses to different antimalarials (shown in the y-axis or below the x-axis). *Indicates significant differences (P < 0.05, Mann–Whitney U test) in sensitivity between parasites carrying the two alleles or haplotypes.

## Slide 4
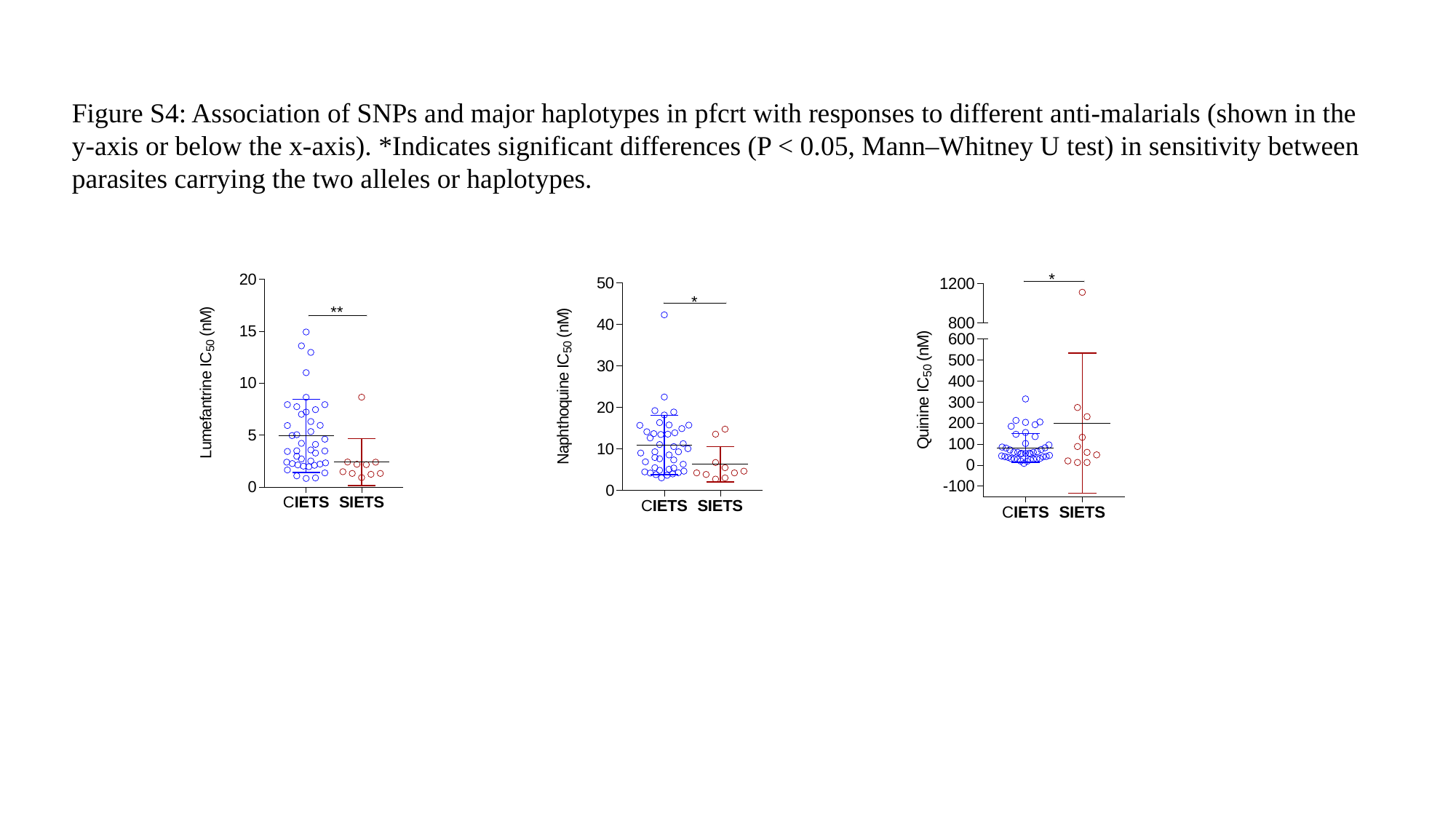

Figure S4: Association of SNPs and major haplotypes in pfcrt with responses to different anti-malarials (shown in the y-axis or below the x-axis). *Indicates significant differences (P < 0.05, Mann–Whitney U test) in sensitivity between parasites carrying the two alleles or haplotypes.

## Slide 5
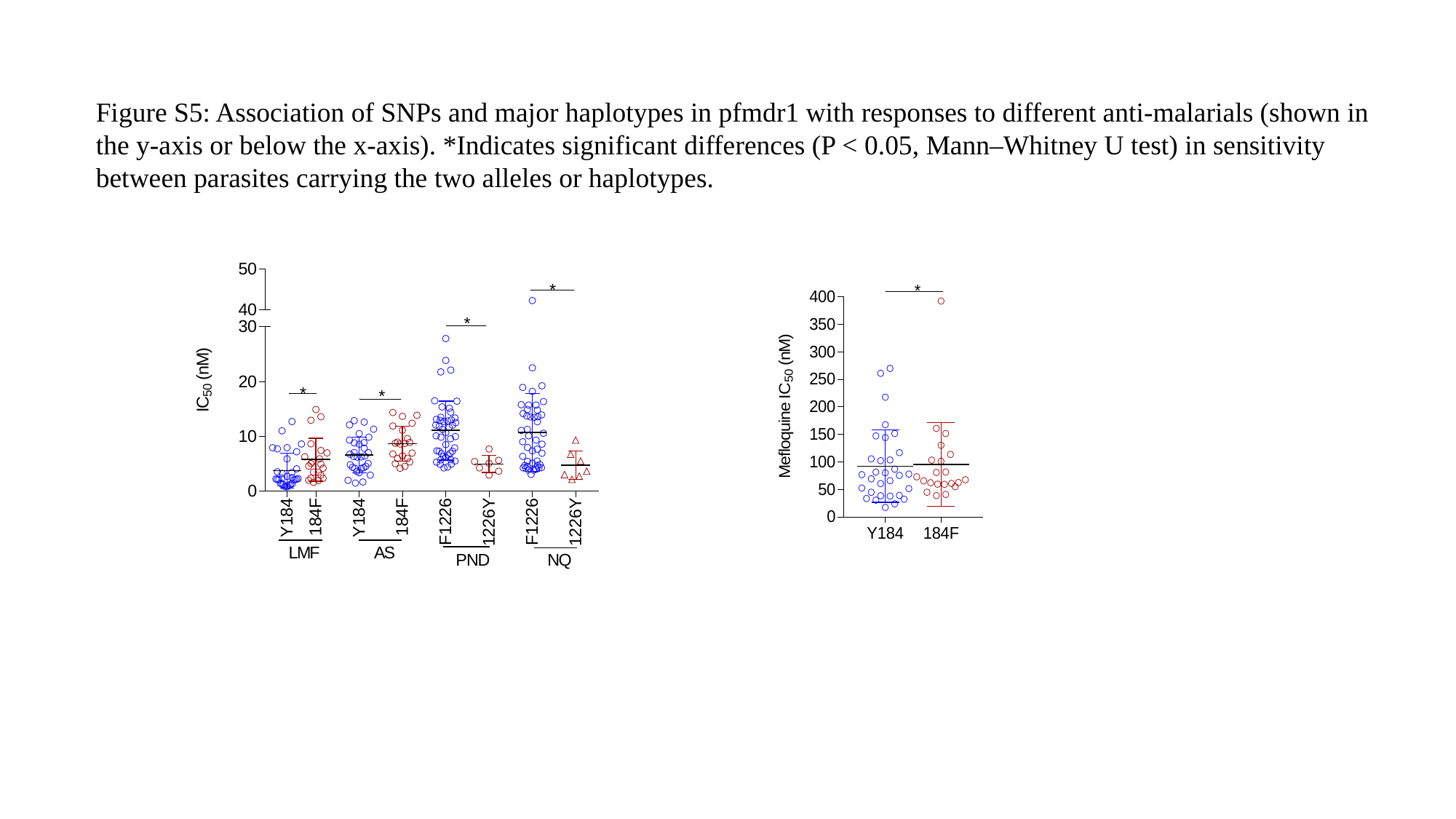

Figure S5: Association of SNPs and major haplotypes in pfmdr1 with responses to different anti-malarials (shown in the y-axis or below the x-axis). *Indicates significant differences (P < 0.05, Mann–Whitney U test) in sensitivity between parasites carrying the two alleles or haplotypes.

## Slide 6
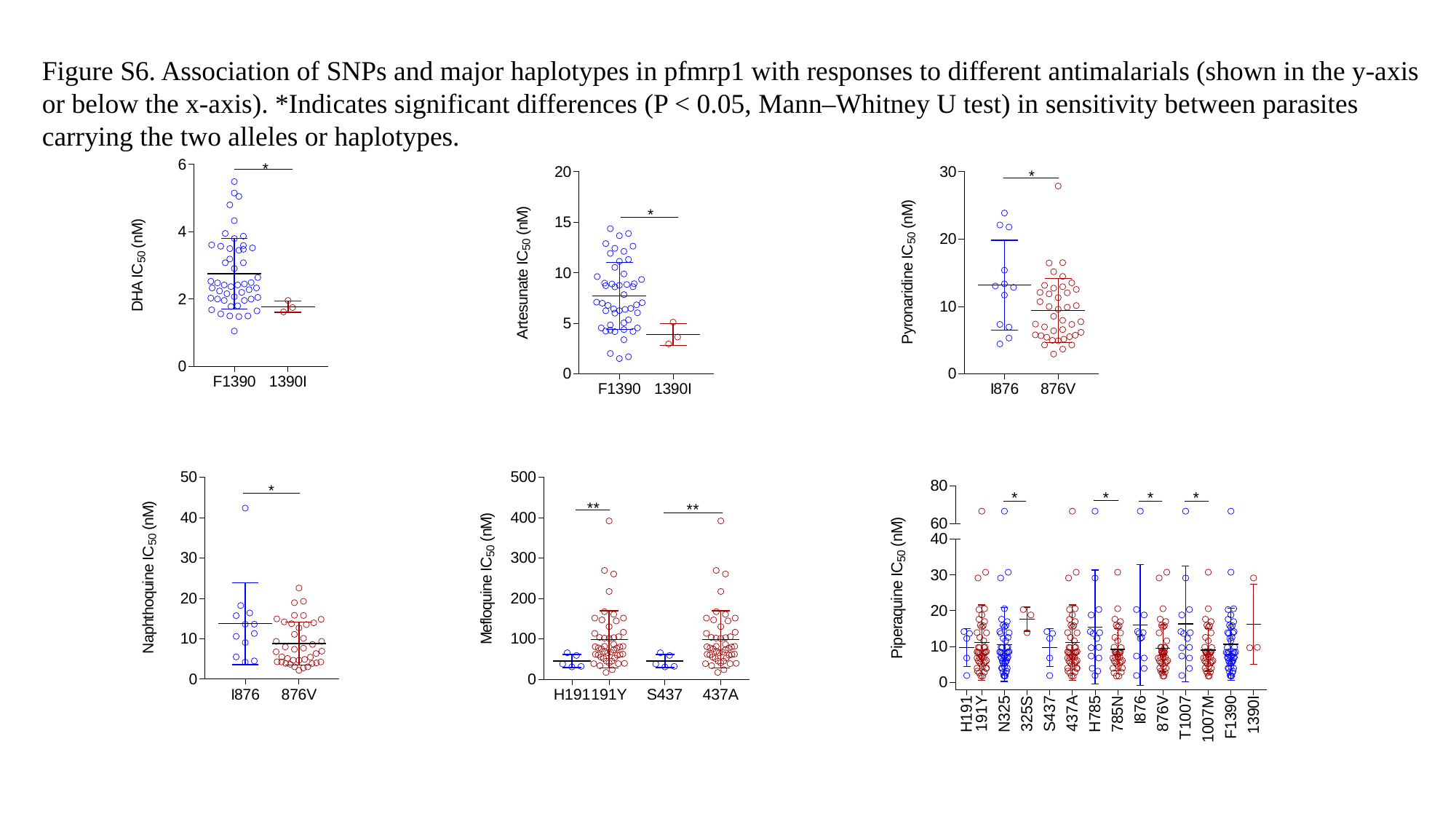

Figure S6. Association of SNPs and major haplotypes in pfmrp1 with responses to different antimalarials (shown in the y-axis or below the x-axis). *Indicates significant differences (P < 0.05, Mann–Whitney U test) in sensitivity between parasites carrying the two alleles or haplotypes.

## Slide 7
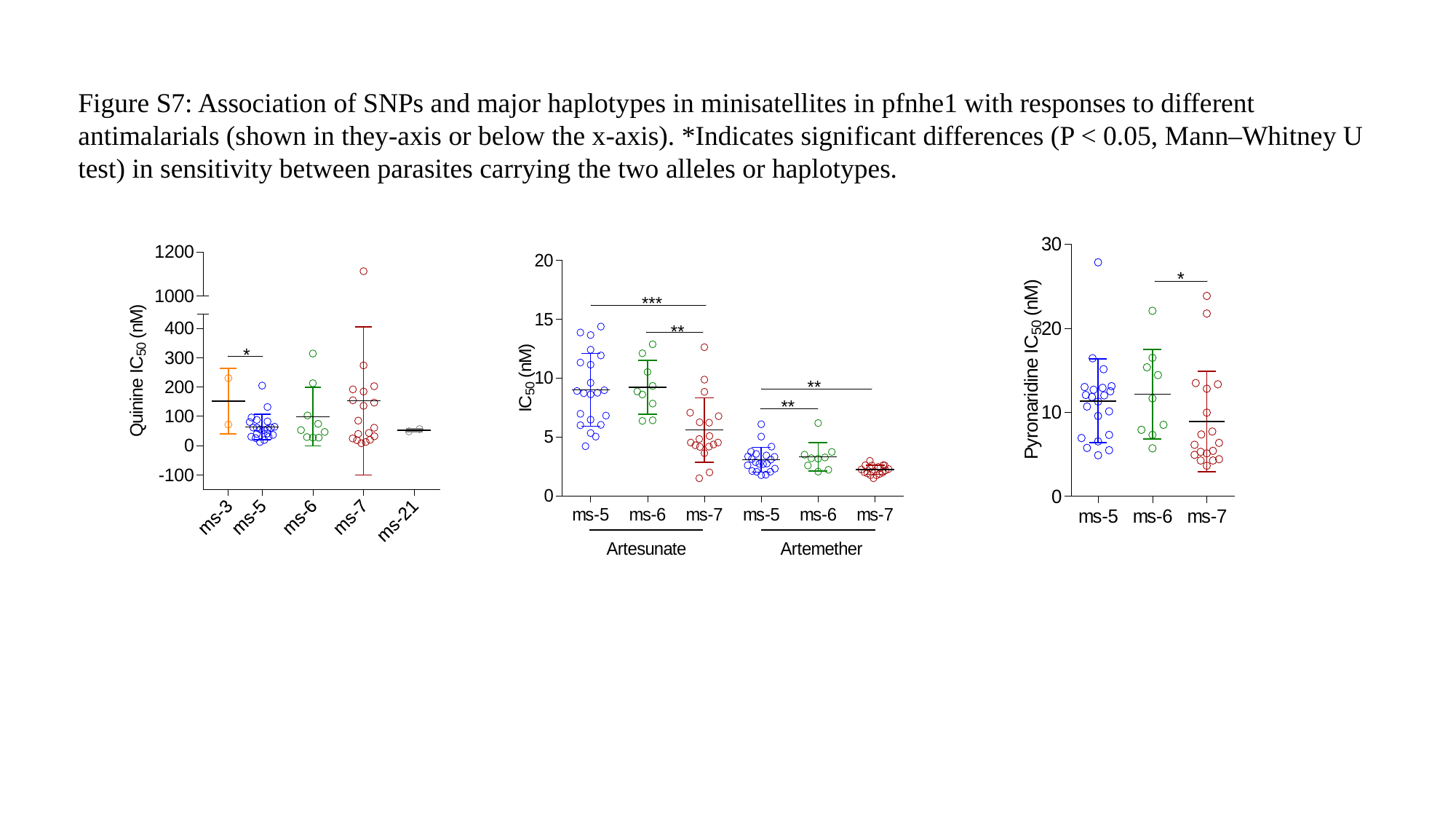

Figure S7: Association of SNPs and major haplotypes in minisatellites in pfnhe1 with responses to different antimalarials (shown in they-axis or below the x-axis). *Indicates significant differences (P < 0.05, Mann–Whitney U test) in sensitivity between parasites carrying the two alleles or haplotypes.

## Slide 8
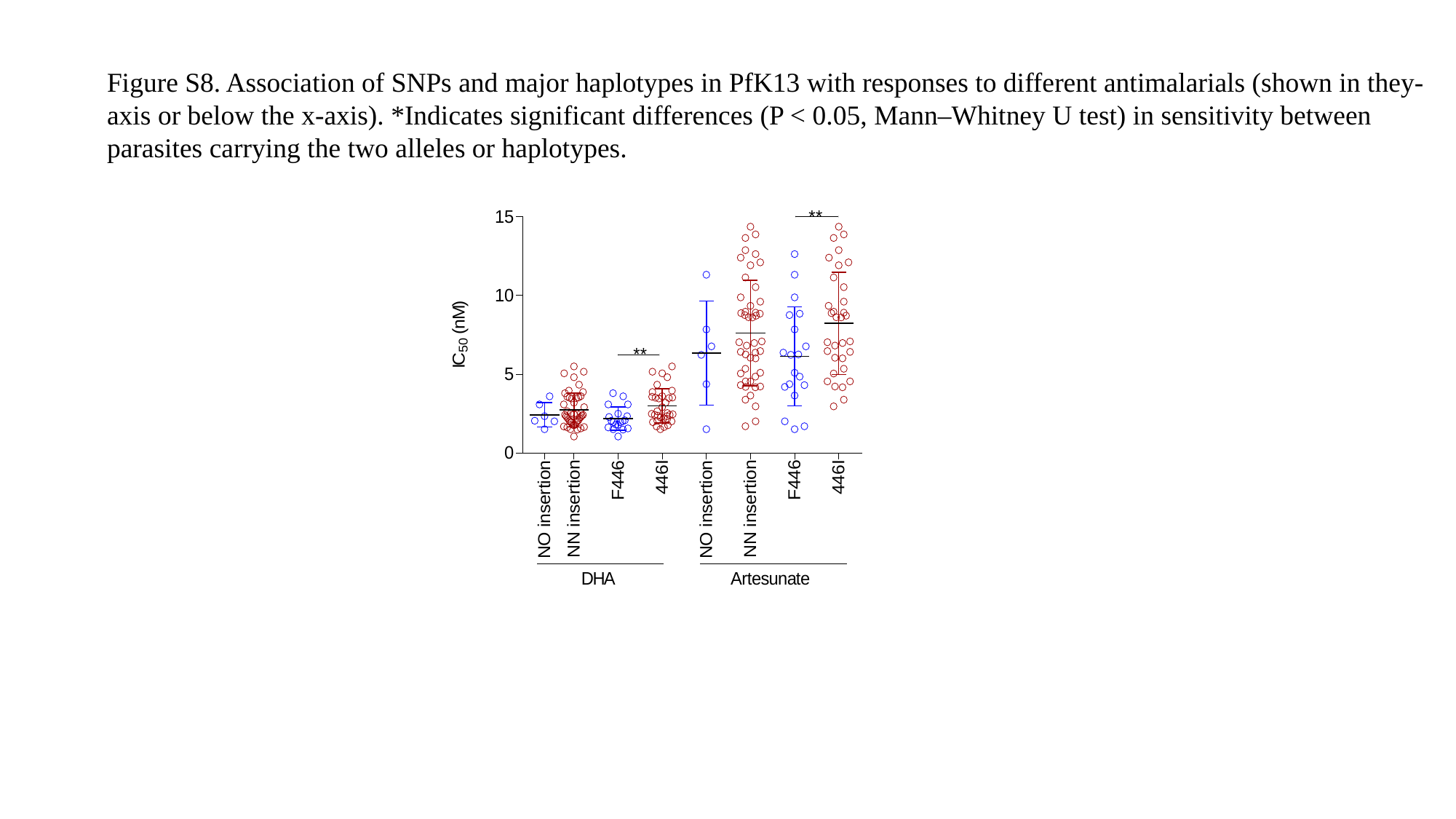

Figure S8. Association of SNPs and major haplotypes in PfK13 with responses to different antimalarials (shown in they-axis or below the x-axis). *Indicates significant differences (P < 0.05, Mann–Whitney U test) in sensitivity between parasites carrying the two alleles or haplotypes.

## Slide 9
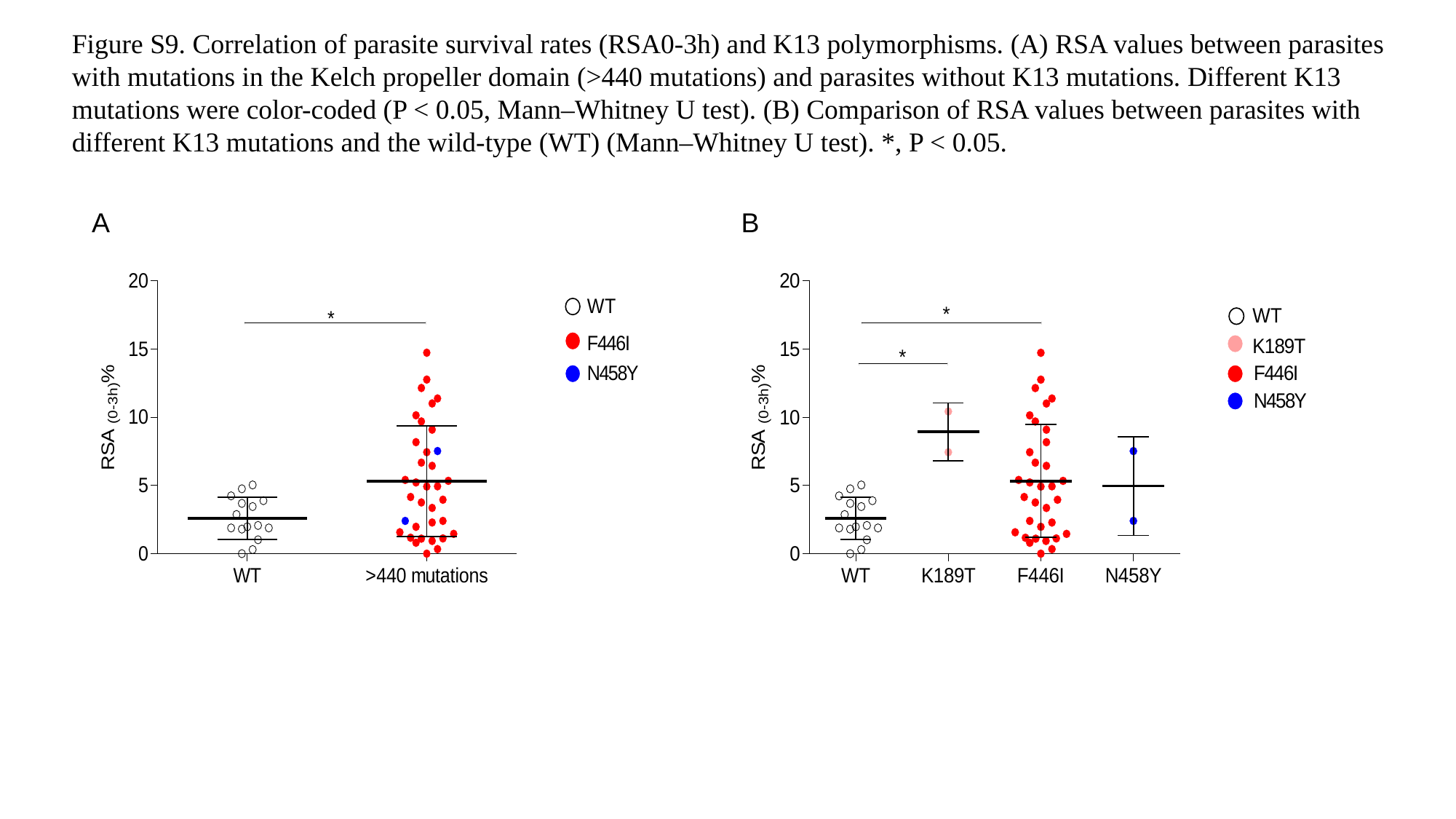

Figure S9. Correlation of parasite survival rates (RSA0-3h) and K13 polymorphisms. (A) RSA values between parasites with mutations in the Kelch propeller domain (>440 mutations) and parasites without K13 mutations. Different K13 mutations were color-coded (P < 0.05, Mann–Whitney U test). (B) Comparison of RSA values between parasites with different K13 mutations and the wild-type (WT) (Mann–Whitney U test). *, P < 0.05.
A
B

## Slide 10
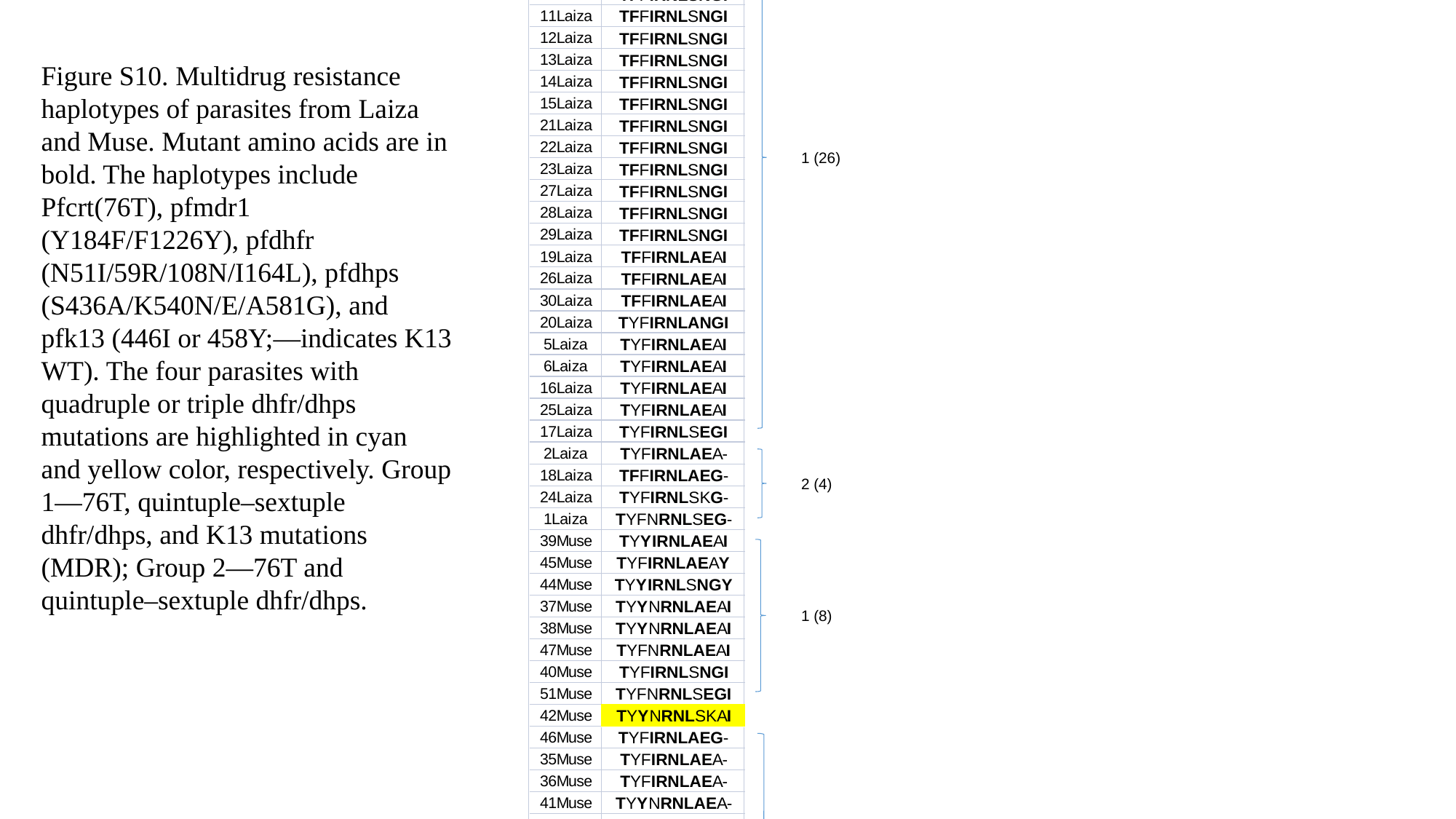

1 (26)
Group (N)
2 (4)
1 (8)
2 (11)
Figure S10. Multidrug resistance haplotypes of parasites from Laiza and Muse. Mutant amino acids are in bold. The haplotypes include Pfcrt(76T), pfmdr1 (Y184F/F1226Y), pfdhfr (N51I/59R/108N/I164L), pfdhps (S436A/K540N/E/A581G), and pfk13 (446I or 458Y;—indicates K13 WT). The four parasites with quadruple or triple dhfr/dhps mutations are highlighted in cyan and yellow color, respectively. Group 1—76T, quintuple–sextuple dhfr/dhps, and K13 mutations (MDR); Group 2—76T and quintuple–sextuple dhfr/dhps.

## Slide 11
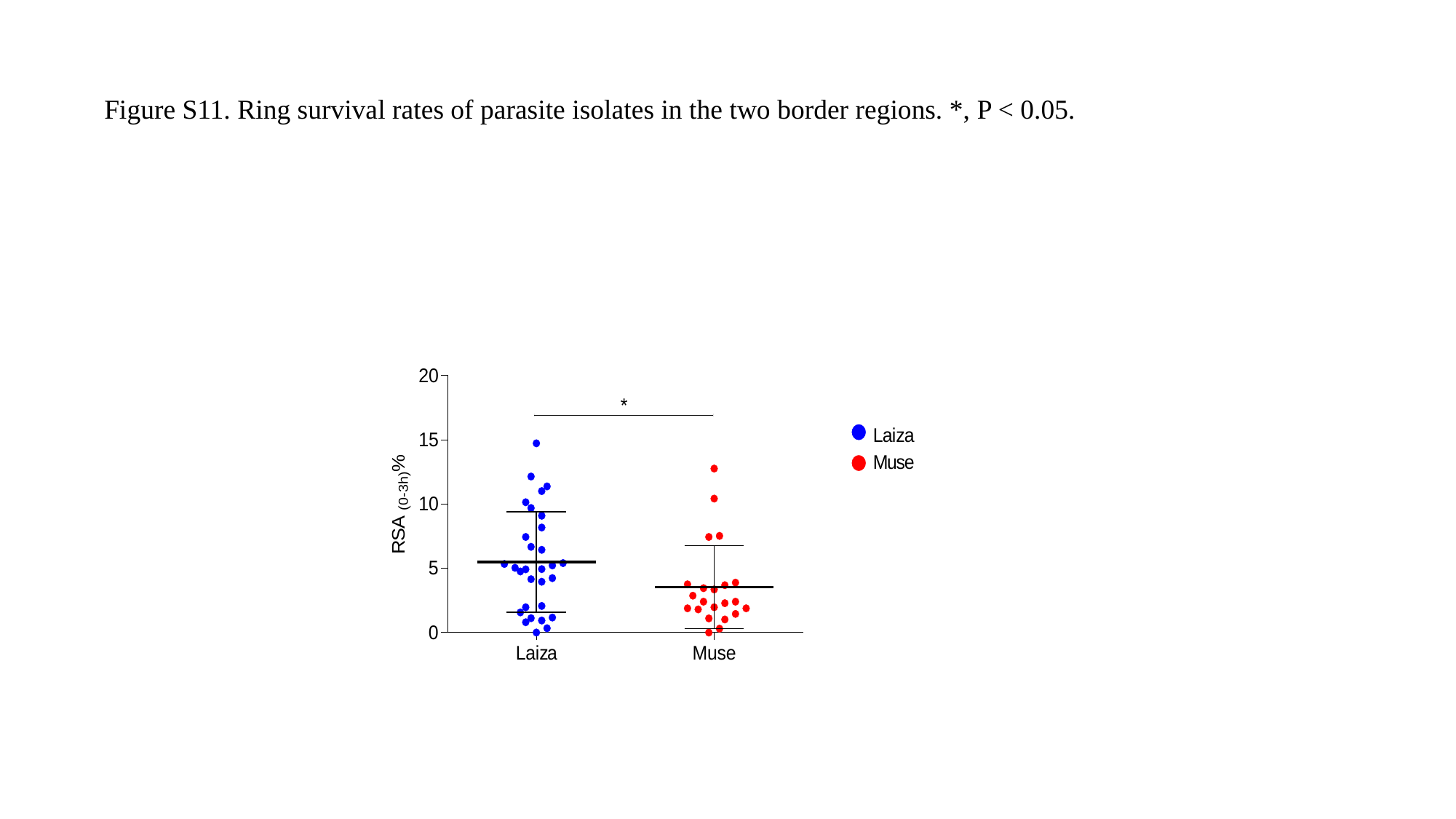

Figure S11. Ring survival rates of parasite isolates in the two border regions. *, P < 0.05.
