# Supplementary material for: Different In Vitro Drug Susceptibility Profile of Plasmodium falciparum Isolates from Two Adjacent Areas of Northeast Myanmar and Molecular Markers for Drug Resistance
Source: Trop Med Infect Dis. 2022 Dec 17;7(12):442. doi: 10.3390/tropicalmed7120442 (PMC9782301; doi:10.3390/tropicalmed7120442)
Supplement: Supplementary file 1 [file tropicalmed-07-00442-s001.zip › Table S2.pdf]

Table S2. Ring survival rates of parasite isolates stratified by the K13 alleles.

Mutations	N	Median RSA value (%)	IQR	P*
WT[#]	15	2.08	1.81-3.89	
K189T	2	8.94	7.45-10.43	0.0147
F446I	33	4.93	1.52-8.63	0.0381
N458Y	2	4.96	2.40-7.52	0.2941

[#] Parasite isolates without any point mutations (including 11 parasites with the NN insertion).
[*] Comparison between the isolates carrying individual K13 mutations with the WT by Mann-Whitney U test.
N, number of parasite isolates; IQR, interquartile range.
